# Feasibility and acceptability of the paediatric pulse oximeter in integrated management of neonatal and childhood illnesses (IMNCI) services by public health facilities: A qualitative study in rural Western India

**DOI:** 10.7189/jogh.13.04105

**Published:** 2023-09-15

**Authors:** Dhiraj Agarwal, Manisha Gore, Anand Kawade, Sudipto Roy, Ashish Bavdekar, Harish Nair, Sanjay Juvekar, Girish Dayma, RESPIRE Collaboration

**Affiliations:** 1 Vadu Rural Health Program, KEM Hospital Research Centre Pune; 2 Symbiosis Community Outreach Programme and Extension, Faculty of Medical and Health Sciences, Symbiosis International Deemed University, Pune; 3 Indian Council of Medical Research, New Delhi; 4 Paediatrics Department, KEM Hospital, Pune; 5 Centre for Global Health, Usher Institute, Edinburgh Medical School, University of Edinburgh; 6 Community Health Research Unit, KEM Hospital Research Centre Pune

## Abstract

**Background:**

Pneumonia contributes to about 15% of child deaths globally, with 20% of the overall deaths occurring in India. Although WHO recommends the use of pulse oximeters (PO) in first-level facilities for early detection of child pneumonia in low- and middle-income countries (LMICs), this has not yet been implemented in India. We aimed to assess the feasibility and acceptability of introducing PO in integrated management of neonatal and childhood illnesses (IMNCI) services at primary health centres (PHC) in the rural Pune district.

**Methods:**

We identified medical officers (MO) and auxiliary nurse midwives (ANM) from six PHCs as study participants due to their involvement in the treatment of children. We developed in-depth interview (IDI) guides for both groups to explore their IMNCI knowledge and attitude towards the program through a qualitative study. We conducted interviews with MOs (n = 6) and ANMs (n = 6) from each PHC. The PO module was added to explore perceptions about its usefulness in diagnosing pneumonia. After baseline assessment, we conducted training sessions on adapted IMNCI services (including PO use) for MOs and ANMs. PO devices were provided at the study PHCs.

**Results:**

At baseline, no PO devices were being used at study PHCs; PHC staff demonstrated satisfactory knowledge about paediatric pneumonia management and demanded refresher IMNCI training. They also felt the need to reiterate the PO use for early diagnosis of pneumonia in children and highlighted the challenges encountered in managing pneumonia at PHCs, such as health system-related challenges and parents’ attitudes towards care seeking. There was positive acceptance of training and PO started to be used immediately in PHCs. There was increased confidence in using PO at endline. PO use in examining symptomatic children increased from 26 to 85%.

**Conclusions:**

Paediatric PO implementation could be integrated successfully at PHC levels; we found pre-implementation training and provision of PO to PHCs to be helpful in achieving this goal. This intervention demonstrated that an algorithm to diagnose pneumonia in children that included PO could improve case management.

Pneumonia is a major cause of under-five mortality worldwide, especially in India [1-3], where despite progress in reducing under-five mortality, pneumonia-related deaths remain a public health concern [4,5]. Integrated management of childhood illness (IMCI) is a recommended approach for managing childhood illnesses, including pneumonia, in low-resource settings [6]. However, the use of pulse oximeters (PO) to measure oxygen saturation in suspected pneumonia cases has not yet been included in IMCI services in India [5,7,8]. Hypoxemia (low oxygen saturation) is a strong predictor of pneumonia mortality [9-11], and measuring oxygen saturation with PO in routine outpatient care for children in low-resource settings could help with early identification, as recommended by the World Health Organization (WHO) [12-14]. The introduction of paediatric PO could reduce neonatal and childhood mortality through the detection of hypoxemia and facilitation of timely referral [15]. However, its availability at the primary care level varies due to cost implications and a lack of perceived need by policymakers and healthcare professionals [6,16]. Implementation studies on the use of PO in primary healthcare settings, particularly in low- and middle-income countries (LMICs) like India, are limited [17,18].

Previous studies have shown that clinical symptoms alone are insufficient for an accurate diagnosis of paediatric pneumonia and the estimation of hypoxia [19]. Manual recording of respiratory rate (RR) can be challenging and may result in the misclassification of symptoms, with chest in-drawing not being adequately recognised. Furthermore, healthcare staff often lack the necessary knowledge for the management of pneumonia [20,21]. These factors can lead to incorrect diagnoses and can impact the effectiveness of treatment [22,23]. The inclusion of paediatric PO in primary healthcare settings could significantly reduce neonatal and childhood mortality by enabling early detection of hypoxemia and timely referral [15]. Studies have demonstrated that PO can identify an additional 20% to 30% of hypoxic children compared to relying solely on clinical signs such as grunting and depressed consciousness, which can be imprecise [24,25].

The implementation of paediatric PO in LMICs has been limited, particularly in India, with few studies conducted in primary healthcare settings [8,12,17,26]. Therefore, there is a need for research that specifically examines the feasibility, acceptability, and operational challenges of integrating paediatric PO into IMNCI services at first-level primary healthcare facilities in rural areas of the Pune district. To address this knowledge gap, we aimed to assess the feasibility, acceptability, and operational challenges of introducing paediatric PO in IMNCI services at first-level primary healthcare facilities in the rural Pune district, in order to improve the understanding of potential benefits and challenges associated with the use of paediatric PO in LMIC settings, specifically in India.

## METHODS

This is a primarily qualitative study, supplemented by secondary quantitative data.

### Study area and population

The study was conducted in the rural administrative blocks of Ambegaon and Junnar in Pune district, located 75-80 km from Pune city, with a combined population of around 625 700. The most common employment among the semi-urban, rural, and tribal inhabitants in this area are agriculture and industrial labour. A primary health centre (PHC) is the first point of contact for health care for the village population; each serves 20 000-30 000 individuals and is staffed by medical officers (MOs), paramedics, and others. We selected six PHCs for the study from a list of all PHCs in the Pune district using purposive sampling; in collaboration with the district health officials from the Ambegaon and Junnar blocks, PHC staff helped the King Edward Memorial Hospital Research Centre (KEMHRC) team with implementing the study. These six PHCs together catered to a population of about 135 000 individuals with approximately 14 000 children under five years of age [27]. Four of these PHCs provide health care services to tribal communities. Based on the mandate and defined responsibilities of the PHC staff, we identified MOs and auxiliary nurse midwives (ANMs) as study participants, as they were key service providers in the treatment of under-five children.

### Study tools development

We used an in-depth interview (IDI) schedule for data collection, developing IDI guides for MOs and ANMs separately to explore their knowledge about and attitudes towards IMNCI. The interview guide was created by the KEMHRC Vadu team and consisted of questions designed to assess participants' knowledge about IMNCI and their perceptions, experiences, and challenges they faced regarding the introduction of paediatric PO in the healthcare system. We piloted the interview tool to validate its effectiveness, clarity, and cultural sensitivity with a small sample. Then, researchers identified and refined issues, assessed interview flow and timing, and trained the two interviewers in the qualitative data collection process. This was done to ensure the validity and reliability of the tool.

### Data collection

We used IDI guides, process mapping guides, and clinical case record forms for data collection. A module on pulse-oximetry was added to understand perceptions about the expected usefulness, acceptability, and effectiveness in diagnosing and classifying pneumonia, alongside questions on operational challenges and facilitators. We further developed process mapping tools and workflow tracking sheets for documenting processes at paediatric outpatient departments of primary health centres. A study team comprising project coordinators (PC) (n = 2) and field research assistants (FRAs) (n = 4-6) were trained in data collection. PCs were specifically trained in qualitative data collection methods; they interviewed the MOs and ANMs in the local Marathi language, as they were adept in its use. We conducted interviews with MOs (n = 6) and ANMs (n = 6) from each PHC. Pre-scheduled appointments of the MOs and ANMs were taken for the interviews. We stopped data collection when saturation was reached.

### Baseline assessment

We conducted a baseline assessment of the six selected PHCs to observe the functioning of the system, IMNCI implementation, documentation practices, and overall workload at the outpatient department (OPD) services for treating under-five children. We also conducted IDI with six MOs and six ANMs.

### Interventions

#### Training

After the baseline assessment, MOs and ANMs were trained on adapted IMNCI services, including PO implementation. The training sessions covered study overview, IMNCI algorithm understanding through visuals, hands-on PO device usage, reading values, IMNCI chart referencing, and proper documentation. Frequent staff transfers led to arranging training for new personnel and offering refresher courses to existing staff, ensuring proficiency in delivering IMNCI services.

#### Introduction of paediatric PO in IMNCI services

Paediatric PO devices (Pulse Oximeter 217; DrTrust, USA) for measuring SpO_2_ in under-five sick children presenting with respiratory symptoms were provided at each of the six PHCs. The study team provided technical support to the PHC staff through regular visits, addressing documentation difficulties, encouraging usage of PO for assessment of under five children with respiratory symptoms, and monitoring adherence to the IMNCI algorithm; this was done without interference with the routine activities in the healthcare system.

### Endline assessment

The study team conducted endline assessment of the six selected PHCs to observe the functioning of the system, IMNCI implementation, documentation practices followed, and overall workload at the OPD services for treating under-five children. Furthermore, IDIs were conducted with six MOs and six ANMs.

### Secondary data collection

We collected baseline information on under-five children seeking healthcare services in the six selected PHCs from March to May 2021 using IMNCI forms. Based on this information, we were able to calculate the number of children with respiratory symptoms and the proportion of cases for which the available adult PO device was used by healthcare staff to determine SpO_2_ saturation. The PO devices were distributed in the last week of May 2021. Data collection continued from June through November 2021 to assess the change in the proportion of usage of PO after procurement of the device. Retrospectively, we obtained the number of under-five children attending OPDs of these six PHCs prior to the start of the study; however, information related to respiratory illness and usage of PO was not available.

### Data management and analysis

We transcribed the interviews from the local language (Marathi), translated them into English, and entered into the MAXQDA software for analysis. We analysed the data using an inductive approach with a thematic framework. Thematic qualitative analysis systematically identifies recurring patterns, themes, and concepts in collected data, providing a deeper understanding of participants' experiences and perspectives. The process involves data familiarisation, coding meaningful units, and organising them into broader themes, yielding a comprehensive narrative and valuable insights. This approach offers flexibility and rigour, enabling rich interpretations from qualitative data. For the quantitative data, we calculated the number of under-five children seeking health care services and proportion of PO devices used in the pre- and post-intervention period was calculated.

### Ethical consideration

The KEM Hospital Research Centre institutional ethics committee (Ref No. KEMHRC/RVM/EC/1312), the Academic and Clinical Central Office for Research and Development (ACCORD), and the Edinburgh Medical School Research Ethics Committee (Ref No. 20-EMREC-014) approved this study. We implemented the study following the National Ethical Guidelines for Biomedical and Health Research involving Human Participants issued by the Indian Council of Medical Research in 2017 and the recent National Guidelines for Ethics Committees Reviewing Biomedical & Health Research During COVID-19 Pandemic issued in 2020. All participants who signed the consent form were included in the study. We also obtained a support letter from the Additional Director of Health Services, Pune district (Ref No. SFWB/KEMHRC research study/2019/82227-28 dated 24/12/2019).

## RESULTS

### Baseline assessment (observations by study team at selected PHCs)

At the baseline assessment, we observed the OPD services and process followed by the PHC staff for under-five children across all six PHCs. Generally, only one MO and one ANM were responsible for handling all the OPD patients, including adult and paediatric populations, resulting in substantial backlogs. The ANM was taking the weight and temperature of all children. The MO was performing a general and systemic physical examination. All under-five children with symptoms of cough and cold were classified as having either an upper respiratory tract infection (URTI) or a lower respiratory tract infection (LRTI). No PO device were being used during the physical examination. The MO documented his/her findings and prescribed treatment on the OPD case paper. There was no separate documentation to suggest that the IMNCI protocol was being followed. The IMNCI charts were not displayed in the OPD room.

### Characteristics of study participants

The MOs were in the age range of 38-59 years with basic educational qualification of Bachelor of Ayurvedic Medicine and Surgery (BAMS) barring one who was Bachelor of Medicine and Surgery (MBBS) (**Table 1**). Their job experience ranged between 12-26 years. The four MOs were from the tribal whereas, two were from the rural PHCs.

**Table 1:** Characteristics of the study participants (qualitative study)

Sr. No	ID	Age	Designation	Education qualification	Number of years in service	PHC
1	MO01	38	MO	Bachelor of Ayurvedic Medicine and Surgery	12	Tribal
2	MO02	45	MO	Bachelor of Ayurvedic Medicine and Surgery	18	Tribal
3	MO03	59	MO	Bachelor of Medicine and Bachelor of Surgery	26	Rural
4	MO04	44	MO	Bachelor of Ayurvedic Medicine and Surgery	12	Tribal
5	MO05	42	MO	Bachelor of Ayurvedic Medicine and Surgery	14	Rural
6	MO06	46	MO	Bachelor of Ayurvedic Medicine and Surgery	18	Tribal
1	ANM01	47	ANM	Higher secondary school completed	25	Tribal
2	ANM02	32	ANM	General Nursing and Midwifery	10	Rural
3	ANM03	57	ANM	Higher secondary school completed	34	Tribal
4	ANM04	45	ANM	Secondary school completed	22	Rural
5	ANM05	30	ANM	Higher secondary school completed	15	Tribal
6	ANM06	30	ANM	Higher secondary school completed	5	Rural

ANM – auxiliary nursing midwifery, MO – medical officer, PHC – primary health care centre

The ANMs were in the age range of 32-57 years, four had completed higher secondary school one had completed secondary school and one had completed a General Nursing and Midwifery (GNM) course. The group represented an equal proportion of respondents from tribal and rural areas respectively. The period of service completed to date was in the range of 5-34 years.

### Baseline themes

**Figure 1:**
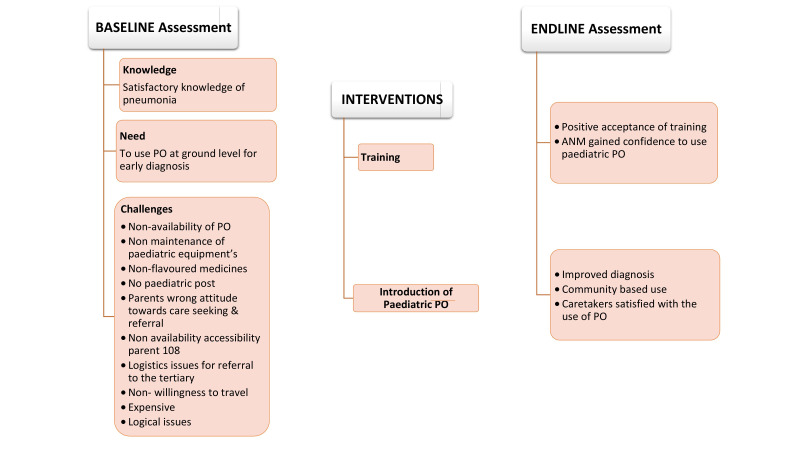
Conceptual framework of the study.

The following baseline themes emerged in the analysis of IDIs both from MOs and ANMs with respect to the feasibility of the introduction of the paediatric PO in the system (**Figure 1**)

#### Theme 1: PHC staff reflected satisfactory knowledge about pneumonia- demanded refresher IMNCI training

Three MOs expressed a desire for refresher training in IMNCI, having completed it previously. They could not use the PO during paediatric examinations due to insufficient equipment.

The ANMs also participated in IMNCI training before and requested a refresher. Their training and work experiences gave them confidence in managing paediatric patients with pneumonia, and they were well-informed about symptoms, clinical presentation, severity grades, and appropriate medical treatments for affected children.

#### Theme 2: The need to reiterate the paediatric PO use and early diagnosis of pneumonia in children was emphasised

The ANMs had not been exposed to the paediatric PO until the interviews. They mentioned that, during the COVID-19 pandemic, they used POs for adults while screening children in the communities. However, the readings consistently showed errors. Since then, they expressed a strong desire to receive hands-on training in using paediatric PO and integrating it into their routine work in the PHCs and communities.

Both ANMs and MOs agreed on the importance of early pneumonia diagnosis in children to prevent complications and avoid referrals to tertiary care hospitals, considering the challenges faced by caregivers in such cases.

#### Theme 3: Challenges faced by participants in the management of childhood illnesses at PHC at baseline and endline

Regarding system-related challenges, MOs and ANMs were disturbed by the lack of paediatric POs and the use of adult equipment for children, which resulted in difficulties fitting children's fingers, battery issues, and incorrect readings. They also expressed concerns about the non-maintenance of other equipment used for treating children, such as paediatric warmers, nebulisers, and emergency kits. Paediatric patients’ parents raised worries about the shortage of medicines and non-flavoured antibiotics causing vomiting in children.

The absence of a paediatric position in the organogram significantly impacted paediatric healthcare. Thus, the MOs and ANMs recommended having a paediatric consultant visits the PHC once or twice a week, along with establishing a separate paediatric facility and maintaining a substantial stock of medicines. They believed that implementing these suggestions would lead to a significant improvement in the outpatient services for paediatric patients at the PHC.

In view of parents’ negative attitude towards seeking health care for their children, the MO and ANM observed that they often displayed negative attitudes towards episodes of cold and cough in children, considering them routine complaints and ignoring the symptoms. They tended to experiment with home remedies and purchase over-the-counter medicines or visit local practitioners, qualified or not, before seeking help from trained clinicians if the symptoms persisted. In critical situations, when the child's health worsens, they agree with the clinician's advice for referral to a tertiary government or private facility, depending on their socio-economic status.

However, many parents feared such referrals, mainly those living in cities, due to limited transportation options and the unavailability of the 108-emergency ambulance service. The expenses incurred for admission, medicines, and other miscellaneous costs exacerbated their concerns. Parents from tribal regions prefer to seek treatment at PHCs as they are financially vulnerable and rely on the free treatment provided at these centres.

### Endline themes

The following endline themes emerged in the analysis of IDIs both from MOs and ANMs with respect to the feasibility of the introduction of the paediatric PO in the system.

#### Theme 1: The training imparted by KEMHRC was positively accepted, resulting in a satisfactory output, and participants expressed satisfaction with the intervention

During the end-line phase, the data revealed that the training organised by KEMHRC received positive feedback and appreciation from the MOs and ANMs. Consequently, they successfully integrated the use of paediatric PO in clinical examinations, enabling regular and efficient patient diagnosis. They were now able to record RR and SpO_2_ of patients with cold and cough symptoms, assess the severity, and provide appropriate treatment. Consequently, there was a significant increase in the number of referrals to tertiary care facilities with paediatricians for further consultation.

The staff expressed satisfaction with the opportunities to apply their training learnings and noticed a visible improvement in patient care. Post-training, the ANMs reported increased confidence and knowledge, leading to the utilisation of POs in community health check-up camps to screen children in villages. Most notably, they started using POs in Anganwadi centres to screen and identify high-risk children under the age of five, suspecting malnourished children to be at higher risk of pneumonia infection.

A few MOs and ANMs were conducting training sessions for accredited social health activist (ASHA) workers in their villages, teaching them how to use the paediatric PO for screening children and making referrals to the PHCs. The ANMs have provided positive feedback about the ASHA workers' involvement, as they showed great motivation in carrying out their outreach duties. This has significantly strengthened the implementation of IMNCI. However, the actual number of patients referred is not reported due to gaps in documentation and record-keeping practices.

#### Theme 2: Confidence in using the paediatric PO was boosted after the intervention

MOs found paediatric PO to be highly useful in the early identification of high-risk children at risk of pneumonia infection. Additionally, parents' appreciation for checking SpO_2_ with the PO has significantly boosted the self-confidence of the MOs and ANMs. Due to increased awareness about children's health post-pandemic, parents now expect SpO_2_ checking in every respiratory clinical examination.

ANMs gained confidence in using the paediatric PO after receiving training. They were able to obtain accurate readings as the children's fingertips fit the sensors perfectly. However, one MO had a differing opinion, which is evident from the verbatim mentioned below.

I disagree. The pediatric PO is not necessary for diagnosing pneumonia in children. Observing physical signs can serve the purpose. A child might have pneumonia even if the oximeter readings are normal. If the child experiences hypoxemia, even the mother can identify the signs without the need for a PO. *– A MO from rural PHC.*

### Quantitative data collection summary

PO (adult device) was used among 26% of paediatric patients with respiratory tract symptoms from March to May 2021, increasing to 8% from June to November 2021 after paediatric PO devices were provided (**Table 2**). However, re-classification of severe pneumonia could not be observed, possibly due to the limited number of under-fives seeking treatment at the PHC level.

**Table 2:** Proportion of usage of pulse oximeters in under five children with respiratory tract infection during pre- and post-intervention

	Prior to intervention of PO (Mar to May 2021)				Post-intervention of PO (June to November 2021)			
**Name of PHC**	**OPD of children <5 y**	**RTI cases**	**PO used in RTI cases**	**Proportion of PO used**	**OPD of children <5 y**	**RTI Cases**	**PO used in RTI Cases**	**Proportion of PO used**
Aptale	103	43	18	0.42	194	111	109	0.98
Madh	51	20	12	0.60	211	82	70	0.85
Sawargaon	76	26	8	0.31	171	94	68	0.72
Taleghar	66	20	1	0.05	195	68	59	0.87
Dimbha	70	38	2	0.05	194	83	67	0.81
M Padwal	34	19	2	0.11	65	31	26	0.84
Total	400	166	43	0.26	1030	468	399	0.85

OPD – outward patient department, PHC – primary health care centre, PO – pulse oximeter, RTI – respiratory tract infections

## DISCUSSION

We found that knowledge and training on the identification and diagnosis of child pneumonia benefitted the healthcare staff at the PHCs significantly. They reported that it refreshed the knowledge they received in IMNCI training previously organised by the government. They became confident in using the PO and were able to reduce the errors in recordings. The proportion of PO use in examining symptomatic children increased from 26% to 85%. Crucially, after the intervention, healthcare professionals began monitoring the RR and SpO_2_ of the children, which fundamentally transformed how patients were examined before the intervention. Second, the ANM-ASHA cadre started implementing the PO in the community and also using it in the Anganwadi centres, which helped improve the screening and referral of pneumonia cases. The team reported various barriers in providing paediatric health care in PHCs and made recommendations for improvement.

The WHO modified its recommendations on the management of pneumonia in healthcare facilities and recommended the use of pulse oximetry, depending on availability [6]. Previously, pulse oximetry was only used for routine screening of all infants born in the early post-natal period (before release from the hospital) in various high-income countries in order to detect critical congenital cardiac disease with high specificity and moderate sensitivity [28]. However, paediatric PO use in low-income countries remains restricted because of various limitations, including the non-availability of paediatric probes, cost, poor maintenance of the devices, lack of training of staff in PO use, inadequate supervision, and others [19-22]. We showed that introducing paediatric PO to assess children for signs of pneumonia in the primary healthcare setting is feasible, with acceptance and support by the parents. The MOs and ANMs were satisfied with the paediatric PO due to its practicality, which is evident from studies conducted in Pakistan and Bangladesh [18,29]. While the Pakistan study showed that SpO_2_ measurements could be taken with a PO in less than one minute and with high parental approval, indicating that it was practical in primary care settings [18], the Bangladesh study demonstrated that PO assessment was successful in 85% in first attempt and in 69% within one minute [29]. The study authors found a 92% (95% confidence interval (CI) = 91-93) adherence to standard operating procedure related to pulse oximetry, and 97% (95% CI = 96-98) agreement in identifying hypoxaemia. The median performance time was 36 seconds (interquartile range (IQR) = 20-75), which was longer among younger children (2-11 months: 44 seconds, IQR = 22-78; 12-59 months: 30 seconds, IQR = 18-53) (*P* < 0.01) and among those classified as pneumonia/severe-pneumonia than as no-pneumonia (2-11 months: 41 seconds, IQR = 22-70; 12-59 months: 32 seconds, IQR = 20-62 (*P* < 0.01) [30]. The finding about staff’s confidence to use PO and positive response by caregivers is corroborated by Bangladesh study [29].

Research studies have shown that using the paediatric PO has made it easier to diagnose severe pneumonia in healthcare centres as well [14,15]. In another observational study, the use of pulse oximetry helped health professionals make better decisions when referring children with severe pneumonia, which is somewhat in line the findings of our study, where we observed increased referrals after the use of PO [30]. Low compliance with referral in the study settings was mostly caused by restricted access to transportation. This suggests that improving treatment outcomes may require more than just providing PO, for example – PO and effective management with the required equipment to diagnose and treat pneumonia. However, our study implies that it is important that the government should consider providing pneumonia treatment at PHCs, which should be investigated in future research [30].

A qualitative study in Bangladesh and Malawi explored the experiences of the use of paediatric PO, and found that health care professionals gained confidence through the use of the PO, gained caregivers’ trust, experienced challenges while implementing it on smaller children and reported battery issues which were similar to the present study [17].

The observation of an overall drop in the paediatric OPD attendance at the six PHCs could be the result of COVID-19-associated restrictions. Some other reasons could be seasonal change, parents' ignorance, and reluctance to bring children to PHCs due to fear of the pandemic [31,32]. According to the ANM, upgrading the PHC with equipment to treat other paediatric illnesses could also aid in boosting OPD attendance.

Our findings about ANM and MO recalling having previously received IMNCI training, but requesting refresher training suggests that a one-time impartment of training is not beneficial unless periodically reiterated. The pre-implementation training helped the healthcare professionals; depending on their training and experience, they may have used their clinical judgment while referring the patients. Additionally, there is a requirement for periodic training, as a few health professionals have not attended the training. The IMCI global survey report reveals that many countries are using an abridged version of the training due to costs incurred and the long absence of healthcare professionals from services. In India, it has been often highlighted that the training targets cannot be fulfilled. Regular technical assistance to the healthcare staff kept them motivated, and routine training and supervision is key for the positive incorporation of PO within primary health care. Timely action should be taken to avoid delays in the treatment and referral of children [17,18,29,30].

The study has several limitations. During the study, the second wave of the COVID-19 pandemic reached its peak, and most public health personnel were assigned to COVID-19 management. Identification and procurement of the appropriate paediatric PO device was a challenge, necessitating significant time and effort from the KEMHRC team. Additionally, the identification of a good, calibrated PO device that is cost-effective, readily available, and sturdy to handle may pose a challenge in scaling up this implementation.

While there was established periodic monitoring during the implementation process, there was no system to scientifically assess whether the providers were accurately diagnosing the conditions. Future research should include a rigorous evaluation. As interviewers were from the national health programmes, they could have expressed opinions favourable to the interviewers.

## CONCLUSIONS

Our findings show that paediatric PO could be integrated at the primary healthcare level successfully. The pre-implementation training and the provision of the device to PHCs were found to be helpful. The fact that paediatric PO is being used more frequently demonstrates that there is a need for the device, and that it is possible to introduce and use it in the healthcare system in a way that could assist in identifying pneumonia in children under five years. The intervention demonstrated that an algorithm including PO to diagnose pneumonia in children could improve case management. The Sustainable Development Goal (SDG) target of reducing under-five mortality will be more achieavable by enhancing case management of pneumonia at the primary care level by expanding ARI diagnostic aids, improving coverage of IMNCI, strengthening referral pathways, and improving the quality of treatment in referral facilities.
